# Rapid Detection of *Plasmodium vivax* by the Hematology Analyzer for Population Screening

**DOI:** 10.3390/diagnostics13223397

**Published:** 2023-11-07

**Authors:** Shanaz Khodaiji, Kunal Sehgal, Monisha Sethi, Dia Mansukhani

**Affiliations:** 1 P.D. Hinduja National Hospital & Medical Research Centre, Mumbai 400016, India; 2 Sehgal Path Lab, Mumbai 400058, India; 3 Sysmex India Private Limited, Mumbai 400078, India; sethi.monisha@sysmex.co.in

**Keywords:** malaria, Sysmex XN, Sysmex XN-L, iRBC flag, *P. vivax*

## Abstract

In India, where malaria is endemic, the prompt and accurate detection of infections is crucial for disease management and vector control. Our study aimed to evaluate the “iRBC” flag, a novel parameter developed for routine hematology analyzers, for its sensitivity and specificity in detecting *Plasmodium vivax* (*P. vivax*) infections. We used residual blood samples from patients with suspected malaria and compared the iRBC flag results with microscopy, which serves as the gold standard. Additionally, we compared the results with rapid immuno-chromatographic tests (RDTs) commonly used in the field. Our study included 575 samples, of which 187 were positive for *P. vivax*. The iRBC flag demonstrated a high sensitivity of 88.7% and 86.1% on the XN and XN-L hematology analyzers, respectively, and a clinical specificity of 100% on both analyzers. Furthermore, the scattergram derived from each positive dataset exhibited distinct patterns, which facilitated rapid confirmation by laboratory specialists. Notably, the iRBC flag remained effective even in the presence of interfering conditions. Overall, our results indicate that the iRBC flag is a reliable and rapid screening tool for identifying *P. vivax* in routine blood testing. Our findings have significant implications for malaria detection and control in endemic regions like India.

## 1. Introduction

Malaria is a significant global health issue, with millions of cases and hundreds of thousands of deaths recorded annually [1]. In 2017, there were approximately 219 million cases of malaria, leading to around 435,000 deaths. Despite efforts to reduce its occurrence, the utilization of diagnostic testing for malaria has increased from 40% in 2010 to 76% in 2015 [2]. The clinical laboratory plays a crucial role in providing a correct diagnosis of malaria for febrile patients. Microscopy, considered the gold standard for malaria diagnosis [3], is subjective and time-consuming and requires a skilled technologist [4]. Since a complete blood count (CBC) is an essential investigation for patients presenting with fever in the emergency department, there is a growing interest in utilizing automated hematology analyzers for malaria detection to provide an early and sensitive indication of infection [5]. The rapid diagnosis of malaria infection is particularly critical in endemic regions.

Previous studies have explored the use of hematology analyzers for malaria detection, employing different principles such as detecting malaria pigment (hemozoin in monocytes), analyzing depolarized laser light diffraction patterns, and detecting increased activation of monocytes [6,7]. These techniques offer rapid checks for possible infections, although they rely on surrogate parameters for malaria detection. On the instrument’s graphical representation, these abnormalities are presented as irregularities in the hematology scattergram, but they require some form of operator training and can be subjective in interpretation [8]. Sysmex hematology analyzers employ fluorescence flow cytometry for blood cell counting and can detect malaria-infected red blood cells while performing a CBC. Infected red blood cells are identified as an additional cluster on the scattergram, generating an “iRBC flag.” This flag indicates red blood cells with inclusions, signifying the presence of parasites in the red blood cells (RBC), which the system automatically recognizes. Without this feature, the additional cell cluster can affect white blood cell differential counts. In essence, malaria detection is based on a specific white cell differential by fluorescence (WDF) scattergram during a 1 min CBC analysis. This is depicted on the side scattering and side fluorescence chart, similar to a conventional flow cytometer, and infected red blood cells appear as an additional cluster overlapping the existing RBC interrogation region. In such cases, the system alerts the user to potential infections with a specific iRBC flag, prompting a slide review for confirmation. Unlike earlier surrogate methods of detection, this approach offers a specific indication of parasite presence in RBCs. Malaria infection can also be confirmed through various other approaches, including quantitative buffy coat (QBC) analysis, immunochromatographic tests, rapid diagnostic tests (RDTs), and polymerase chain reaction (PCR) assays. While PCR demonstrates superior sensitivity and specificity compared with RDT and microscopy [9], its utility is limited due to labor-intensive sample processing, relatively high costs, and the need for specially trained technologists [10]. To provide value-based healthcare at the institution, efficient diagnostic tests should meet criteria like adequate specificity and sensitivity, ease of performance with a satisfactory turnaround time (TAT), and a reasonable cost [11]. Hematology analyzers offer a potentially efficient diagnostic tool as they require no additional cost when a CBC is ordered, and the turnaround time is approximately 1 min per blood test.

As part of our value-driven outcomes analysis, our study aims to evaluate the effectiveness of the iRBC flag on two commonly available routine hematology analyzers (XN and XN-L) in detecting *P. vivax* malaria, comparing it with microscopy and rapid diagnostic tests (RDT). The study also takes into consideration potential interfering factors such as anemia, reticulocytosis, thrombocytopenia, eosinophilia, and dengue infections to rule out potential confounding conditions commonly seen in patients seeking medical attention at our facility.

## 2. Materials and Methods

### 2.1. Study Design and Sample Collection

This was an observational study, where samples were collected within a 6-month period. Approval was provided by the Research and Ethics Committee of the P.D. Hinduja National Hospital and Medical Research Centre, Mumbai, India (872-14-SK/KS), and a waiver for consent was granted. The microscopy and RDT were carried out on all consecutive samples referred to the laboratory for malaria parasite detection. All blood samples were collected in K2-EDTA vacutainers. Samples that tested positive for *P. vivax* using both methods were included in the study. Samples with discrepant results or that tested negative using both methods were excluded from the study. In order to address the operational efficiency of the hematology analyzer for *P. vivax*, samples with other parasite species or mixed infections were excluded. In order to ensure blood conditions were optimal during testing, aged samples greater than 8 h were also excluded. Finally, to account for potential interfering factors, we included various negative samples with specific conditions as shown in Table 1.

### 2.2. Hematology Analyzer Settings for iRBC Flagging

The hematology diagnostic tests were conducted on the Sysmex XN and XN-L analyzers (Sysmex Corp., Kobe, Japan). In the XN analyzer, both the white count and nucleated red blood cells (WNR) channel (SFL-FSC axis) and the WDF channel (SSC-SFL axis) were used to detect the presence of *P. vivax* malaria-infected RBCs, whereas only the WDF channel was used in the XN-L analyzer. These infected RBCs were visualized as a cluster of particles in the flow scattergram. The iRBC flag triggered internally by the system when the levels were above 100 arbitrary units (AU), with a scale up to 300 AU. The iRBC flag levels associated with both samples showing positive parasite infections and control samples were examined. These samples were deemed highly suspicious for positive malaria infection. The result was automatically displayed on the instrument touchscreen associated with the sample. All samples were processed on the XN analyzer in CBC+DIFF+RET+PLT-F+WPC mode and on XN-L in CBC+DIFF+RET mode to look for the iRBC flag for *P. vivax* detection.

### 2.3. Reference Method for Malaria Confirmation

Microscopic slides were reviewed for *P. vivax* detection by two trained microscopists independently. Thin films were examined under 100× magnification to estimate the *P. vivax* parasite count/infestation rate (IR) using the formula

% Parasitemia = number of parasitized RBC (3000 RBC were counted) × 100/3000

The IR refers to the density of positively identified infected RBCs within a patient sample. A total of 3000 RBCs were assessed, and the result was expressed as a percentage shown using % parasitemia. This measure correlated to cases of high or low parasitemia load within the patient.

RDT was performed by using the SureTest MAL Malaria pLDH/HRP2 combo test kit (MicroGene Diagnostic Systems (P) Ltd., New Delhi, India). The sensitivity and specificity of the iRBC flag from the hematology analyzer were determined by comparison with microscopy (gold standard) and RDT.

### 2.4. Statistical Methods

The sensitivity and specificity of hematology assays for detecting malaria were evaluated using the results obtained from either the XN or the XN-L instrument. These results were then compared with reference methods in the laboratory. Samples that displayed positive iRBC flags in the blood test were examined for correlation with the specific malaria species detected. To assess the accuracy of both instruments, a receiver operating characteristics (ROC) curve was constructed based on the iRBC flag score, using all clinical samples tested on the analyzers. The area under the ROC curve (AUROC) was calculated. All statistical analyses were conducted using MedCalc software version 21 (MedCalc Software Ltd., Ostend, Belgium).

## 3. Results

### 3.1. Study Design

**Figure 1:**
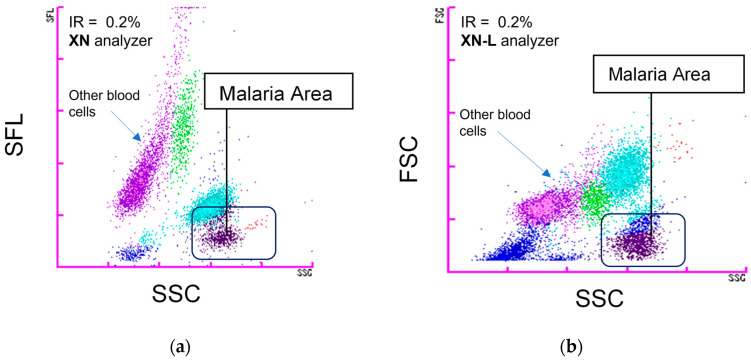
Flow cytometry scattergram plots of blood specimens for samples with high malaria infestation rate (IR). (**a**) Scattergram of a *P. vivax* malaria-positive sample on the XN analyzer (IR 2.0%) showing a parasitized RBC population. A positive iRBC flag is seen on the scattergram in this case. (**b**) Scattergram of a *P. vivax* malaria-positive sample on the XN-L analyzer (IR 2.0%) showing a parasitized RBC population that triggers the iRBC flag.

The study included a total of 187 samples that tested positive for *P. vivax* using both microscopy and RDT to determine the diagnostic sensitivity. Among the positive samples, 164 had an IR of ≥0.1%, while 23 samples had an IR of <0.1%. Samples with low IR are frequent challenges for the even trained microscopist. In addition, 385 negative samples, also confirmed by both microscopy and RDT, were selected to assess the effects of potential interfering conditions, which were categorized in Table 1. The categories included the most commonly detected conditions that may affect the results, such as anemia, reticulocytosis, thrombocytopenia, eosinophilia, and dengue virus infection. The experimental control arm included volunteers from health screenings and randomly selected febrile cases seeking medical attention at the hospital. All 572 samples were processed on both the XN and XN-L hematology analyzers simultaneously to evaluate the effectiveness of the iRBC flags for detecting malaria. The differences between the two instruments were observed in the scattergrams generated by a complete blood count to identify the parasite-infected cells, as illustrated in Figure 1. All other instrument settings remained the same to facilitate comparisons.

### 3.2. Sensitivity and Specificity for P. vivax Detection

The positive detection rate shown on the XN analyzer was 166 of 187 *P. vivax*-positive samples. On the XN-L, 161 samples were positively identified through the iRBC flag indicating the presence of *P. vivax*, and 26 samples were negative (Table 2). Using the iRBC flag score from positive and control specimens, we observed AUROC values of 0.944 for the XN system and 0.930 for the XN-L system in correctly identifying infected samples (Supplementary Figure S1). These data indicated a slightly superior performance of the XN system over the XN-L system for detecting malaria infections. Both instruments, however, exhibited exceptional diagnostic capabilities, making them valuable tools for expediting the screening of malaria infections. In both instruments, the sample turnaround time to obtain a result was 1 min per sample and was performed automatically by the instrument without further intervention. The addition of this positive iRBC flag will greatly streamline laboratory processes and aid in the rapid identification of potentially infected cases, especially in remote regions. Representative flow cytometry scattergram plots are provided in Figure 1. In addition to the sample flags by the instrument to alert healthcare professionals, the scattergram provides a direct visualization of the presence of infected erythrocytes. This is captured at the bottom right corner of each individual scattergram (Figure 1). These distinctive features allow laboratory specialists to quickly identify samples and prioritize for slide preparation and confirmation. The pattern within each scattergram differs slightly among both instruments as highlighted in Figure 1, due to different interrogation channels of the analyzers. Nonetheless, the unique cell population in malaria-positive cases shows how sensitive these hematology instruments are to slight variations in red cell sizes due to parasite infestations. The size changes are represented as a significant displacement from the x-axis of the scatterplot from normal erythrocytes.

For clinical specificity, all 385 samples tested for the effects of interfering conditions did not show the iRBC flag on both the XN and XN-L hematology analyzers, indicating that there were no false positive results in this extensive dataset. Collectively, as summarized in Table 3, the XN analyzer could detect *P. vivax* malaria infection with an overall sensitivity of 88.8% and accompanying specificity of 100%. On the XN-L analyzer, a clinical sensitivity of 86.1% and specificity of 100% were achieved.

### 3.3. Scattergram Patterns for Effective Malaria Detection Workflow

**Figure 2:**
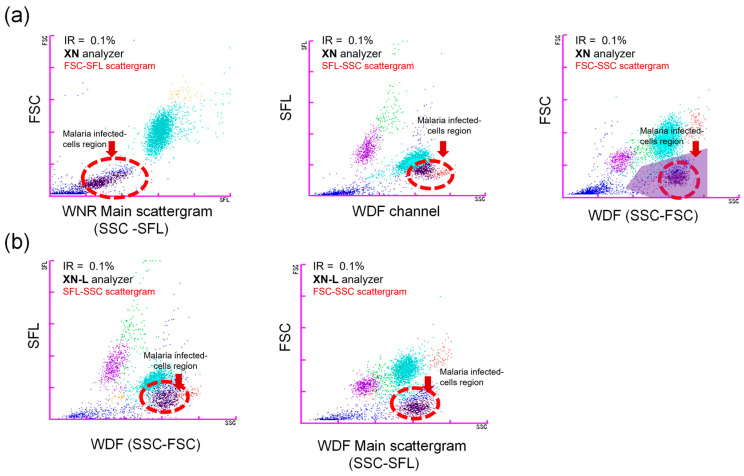
Flow cytometry scattergram plots of blood specimens for samples with a low malaria infestation rate (IR). Typically used scattergrams are the SFL-SSC and FSC-SSC flow plots on the XN and XN-L analyzers, respectively. (**a**) Different scattergrams produced by the XN analyzer. *P. vivax* malaria-positive clustering on the XN analyzer (IR 0.1%) was observed in both WNR and WDF channels. (**b**) Scattergram of a *P. vivax* malaria-positive sample on the XN-L analyzer (IR 0.1%) in the WDF channel.

To ensure low parasitemia cases are thoroughly investigated, specific samples at IR <0.1% were analyzed. At lower infestation rates, we observed the scattergrams to be of lesser intensity but nonetheless distinctive as shown in Figure 2. In difficult cases, where the instrument may not be able to differentiate or does not meet the system threshold for triggering the iRBC flag, having a thorough understanding of scattergram interpretation may serve well as an early indicator for skilled specialists. Subsequent slide preparation and confirmation can be provided for these cases. This complements current laboratory workflows to prioritize samples for laboratory confirmation using microscopy.

Within this dataset, we observed an interesting trend in the IR and positive iRBC detection rates from both analyzers. Samples with low IR showed a reduced clinical sensitivity for the XN and XN-L analyzers as shown in Table 2 using the iRBC flags alone. The sensitivity improved considerably with samples that had a higher IR of more than 0.1%. In analyzing this group, the sensitivity was 93.9% and 91.5% with the XN and XN-L analyzers, respectively. Patients with higher IR have a greater likelihood of developing severe malaria; hence, it is important that these cases are identified early. To highlight the differences in scattergrams results, *P. vivax* malaria samples with low IR (<0.1%) are shown in Figure 2a,b. It is noteworthy to mention that in our observations, all control samples did not show a population of parasitized RBCs on the scattergrams, and the iRBC flags were concurrently negative. As no false positive iRBC flag was generated in this study from our interference experiments, we infer that the potentially interfering factors that were studied, including samples from dengue patients, do not interfere with *P. vivax* detection on the hematology analyzers. From these instrument raw data plots, which are conveniently displayed and stored with each sample run, laboratory specialists can easily assess the patient sample conditions and confirm with the reference methods. This can also quickly aid a pathologist in offline settings to be alert of any potentially positive samples.

## 4. Discussion

India accounts for 77% of all malaria cases in South Asia [12]. However, there is a significant gap (68–95%) between reported and actual malaria cases due to inadequate surveillance and under-reporting [12]. Severe vivax malaria epidemics in Gujarat, India, contribute substantially to the burden of vivax malaria and occur more frequently than falciparum malaria [13]. Delays in the diagnosis and treatment are a leading cause of malaria-related deaths worldwide, emphasizing the need to focus on *P. vivax* detection in this study.

Over the last decade, global malaria incidence has declined due to increased funding for elimination efforts. This shift means that patients presenting with fever are just as likely to have a non-malarial cause for their symptoms. Therefore, confirming malaria accurately, while ruling out other infections, is increasingly crucial to prevent incorrect treatment, unnecessary medication, and the development of drug-resistant strains. Additionally, the proportion of malaria cases due to *P. vivax* is increasing, possibly due to reporting practices and the ability to identify and treat infected patients [14]. This can be due to reporting practices, the ability to identify and treat infected patients, transmission dynamics, etc. [14]. Notably, RDTs have lower sensitivity for *P. vivax* compared with *Plasmodium falciparum* (*P. falciparum*) [15]. In the midst of these challenges, we observed that the iRBC flags in the hematology results were not affected, mainly due to significant changes in erythrocytes during parasitic infections. These changes alter flow scattergram patterns and aid in detecting malaria cases. Our study confirmed the effectiveness of both XN and XN-L analyzers, which cater to both high-volume and smaller clinics, in screening for potential cases, with high AUROC values (see Supplementary Figure S1). The iRBC flag functions as a system alert, signaling the potential presence of an infection, while IR quantifies the concentration of infected red blood cells within each sample. Our research demonstrated the instrument’s sensitivity, even in cases with low parasitemia, by effectively evaluating the IR.

CBC tests are typically conducted in patients with febrile illnesses, providing an excellent opportunity for a timely malaria diagnosis. Technological advancements have improved blood cell type discrimination accuracy, making CBC tests a rapid yet sensitive method for diagnosing and monitoring various clinical conditions. For instance, by addressing activation and increase in white cell differentials, accurate profiling of septic patients in the intensive care unit can be achieved [16]. Until the early 1990s, several malaria-related alterations in the CBC were described, such as abnormal extra peaks in the WBC histograms of a Coulter MaxM analyzer and pseudo-reticulocytosis in a Sysmex R-1000 analyzer [17]. On the Technicon H1 analyzer, malaria-positive samples showed the presence of ≥3% “large unstained cells” (LUCs), the normal range being 3.3–20.9%, suggesting their potential use for malaria screening [17]. Another study carried out by Mukry et al., found several abnormalities in the scattergram of the XE 2100 hematology analyzer in patients with suspected malarial infection. These include pseudo-eosinophilia; graying of the neutrophil cluster and double neutrophil and eosinophil populations in the DIFF channel; more than seven dots along the *x*-axis between the first and third vertical markings (in *P. vivax* infection only) in the WBC/BASO channel; increased signals in the basophil region in approximately all cases of *P. vivax*, whereas basophilia was not seen on the smear; and multiple gray dots in the IMI channel despite the absence of immature granulocytes [18]. These abnormalities supported 99.2% parasite detection, whereas only 93% of confirmed malaria cases were detected by the two rapid tests used in the study. Since the sensitivity of malaria detection was high on the Sysmex XE-2100 analyzer, it can be considered a good option for the presumptive diagnosis of malarial parasites in endemic areas. The authors conclude that microscopy remains the gold standard to confirm malarial parasites in suspected patients. A recent Indian study examined the Sysmex XN-1000 analyzer in malaria detection and found distinctive clusters in the WNR (SFL-SSC) and WNR (SSC-FSC) scattergrams [7]. In addition, the gated events showed a correlation with parasite load, demonstrating another function of the Sysmex analyzer. In a similar work, Huh et al. focused on *P. vivax* cases utilizing the XN-9000 analyzer, commonly found in large hospitals with a heavy CBC workload [19]. In our study, we assessed both high- and low-throughput instruments, namely, the XN and its counterpart, the XN-L, using a paired sample approach to establish the clinical relevance and provide direct comparisons. It is worth noting that in remote and suburban regions, smaller clinics and hospitals predominantly employ the XN-L, where the burden of malaria infection is higher. Consequently, there is substantial value in developing capabilities within these healthcare institutions to address potential early outbreaks. In other related instruments, Pillay et al. used the XN-30 analyzer for malaria detection and results correlated with microscopy, with a sensitivity and specificity of 100% [11]. A French study used the XE-2100 and XE-5000 analyzers to detect malaria from patients who returned from endemic regions [20]. More recently, Zuluaga et al., using the XN-31 instrument, a specialized analyzer for malaria detection, observed strong and compelling data [21]. While it holds potential to greatly support national surveillance or screening programs for malaria, it is not widely accessible within standard healthcare institutions. This limitation restricts its integration into routine care.

These prior studies provided strong evidence endorsing the use of both the XN and XN-L instruments’ iRBC alert for detecting malaria. Our current investigation contributes additional field evidence, reaffirming its effectiveness in identifying samples with low parasite counts. This bears significant importance since cases of low parasitemia can frequently occur, particularly when clinical symptoms are mild. In addition, this study marks the inaugural attempt to meticulously profile and juxtapose the capabilities of both XN and XN-L analyzers for the detection of *P. vivax* malaria, instruments frequently encountered in standard hospital laboratories. To the best of our knowledge, this is also the first study to encompass a wide array of potentially interfering conditions. In our study, the sensitivity for *P. vivax* malaria detection was 88.8% on the XN analyzer and 86.1% on the XN-L analyzer, which is comparable to the study by Mukry et al. The specificity was 100% on both hematology analyzers. We further analyzed the effects of IR in samples. We observed that the sensitivity was directly related to the IR, and improved detection rates were associated with higher IR. Further improvements to detection are possible by finetuning the flagging sensitivity or direct visualization of the scattergram, which can be achieved with the preliminary data from this study. In this current exploratory work, the positive detection of *P. vivax* was based on the clustering of parasitized RBCs in the WDF and WNR channels of the XN and XN-L hematology analyzers. The lower-end XN-L analyzers are typically found in remote regions, and this will greatly enhance the ability for thorough population screening and early detection of malaria outbreaks.

To ensure this is relevant to the actual needs in the field, we profiled samples of potentially interfering conditions, which was novel for this research. Significantly, the iRBC flag was not generated in any of these samples. In view of the similarity in the clinical presentations of dengue and malaria, we included dengue samples in order to rule out the possibility of false positive results due to dengue, hence avoiding a misdiagnosis and its consequences. The results are encouraging, with 100% specificity for all controls. One limitation of the current study is the small sample size for patients’ samples with low IR. We postulate that a larger sample size with diverse IR conditions needs to be tested to ensure the correct and accurate subclass generation and potentially can be used to justify adjusting the flagging sensitivity. We are positively motivated by samples with high IR showing diagnostic sensitivities above 90% as these cases may develop into severe malaria. Another limitation is that this study addressed only *P. vivax*. Further work is required to extend to other pathogenic malarial species like *P. falciparum*, *Plasmodium ovale*, *Plasmodium malariae*, and *Plasmodium knowlesi*. The main focus on *P. vivax* in this study was because this species is the dominant malaria parasite in our region. A significant aspect of our study is the demonstration that the iRBC flag is a good screening tool for the detection of *P. vivax* with potential integration into remote and secondary healthcare facilities equipped with routine hematology analyzers.

## 5. Conclusions

In conclusion, our use of fluorescence flow cytometry has demonstrated remarkable diagnostic sensitivity and specificity in identifying RBCs infected with the *P. vivax* malaria parasite. This method’s direct detection of the parasite not only enhances detection accuracy but also mitigates the issue of false positives associated with alternative approaches. Given the mandatory nature of CBC testing in febrile patients, a simultaneous malaria diagnosis represents a significant advancement in both malaria detection and timely treatment. Moreover, it promises to streamline laboratory practices by reducing the need for unnecessary slide smears. With the added benefits of no additional cost and rapid turnaround times, this method emerges as an excellent first-line screening tool, especially in malaria-endemic regions.

## Figures and Tables

**Table 1:** Selection criteria and number of samples analyzed for the effect of interfering factors in *P. vivax* malaria diagnosis on XN and XN-L hematology analyzers.

Sample Type	Sample Size (*n*)	Inclusion Criteria
Anemia	50	Hb less than 10 g/dL
Reticulocytosis	30	Reticulocyte count more than 5.0%
Thrombocytopenia	42	Platelet count less than 100,000/µL
Eosinophilia	30	Eosinophil count of >1000 cells/µL
Random	100	Five randomly picked samples were run with every batch of patient samples
Healthy	98	From health checkup patients after excluding any co-morbidities
Dengue	35	Dengue samples confirmed by NS1 and dengue serology
Total	385	

**Table 2:** Sensitivity and specificity of iRBC flag for *P. vivax* detection.

Positive by Microscopy and RDT	Positive on XN	Positive on XN-L	Negative on XN	Negative on XN-L
187	166	161	21	26
**Negative by Microscopy and RDT**	**Positive on** **XN**	**Positive on** **XN-L**	**Negative on** **XN**	**Negative on** **XN-L**
385	0	0	385	385

**Table 3:** Sensitivity of *P. vivax* detection in samples with high IR (>0.1%) and low IR (<0.1%) and overall sensitivity and specificity on XN and XN-L analyzers (*n* = 187).

*P. vivax* IR (Parasite %) by Microscopy	Positive (XN)	Positive (XN-L)	Negative (XN)	Negative (XN-L)	Sensitivity (XN)	Sensitivity (XN-L)
≥0.1% (*n* = 164)	154	150	10	14	93.9%	91.5%
<0.1% (*n* = 23)	12	11	11	12	52.2%	47.8%
Total *P. vivax* positive (*n* = 187)	166	161	21	26	88.8%	86.1%

## Data Availability

The data presented in this study are available on request from the corresponding author. The data are not publicly available due to data privacy.

## Supplementary Materials

The following supporting information can be downloaded at https://www.mdpi.com/article/10.3390/diagnostics13223397/s1: Figure S1: Receiver operating characteristics curve to compare results of identifying malaria infected samples using the XN and XN-L instruments.
