# Supplementary material for: Rapid Detection of Plasmodium vivax by the Hematology Analyzer for Population Screening
Source: Diagnostics (Basel). 2023 Nov 7;13(22):3397. doi: 10.3390/diagnostics13223397 (PMC10670845; doi:10.3390/diagnostics13223397)
Supplement: Supplementary file 1 [file diagnostics-13-03397-s001.zip › diagnostics-2617991-supplementary.pdf]

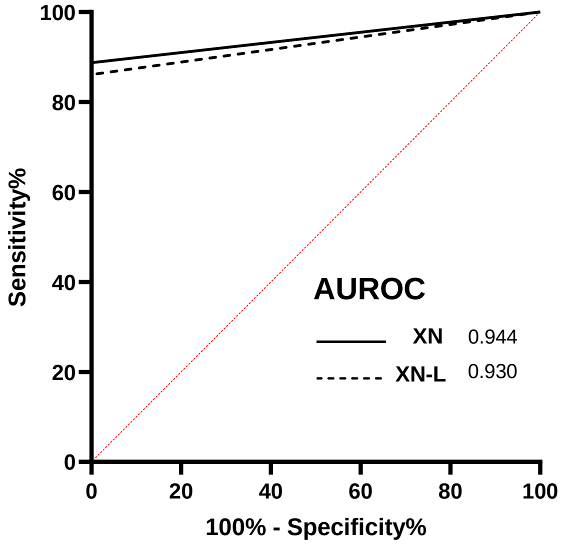

Supplementary Figure S1. Receiver operating characteristics curve to compare results of identifying malaria infected samples using the XN and XN-L instruments.
